# Anticontrol of Hopf Bifurcation and Control of Chaos for a Finance System through Washout Filters with Time Delay

**DOI:** 10.1155/2014/983034

**Published:** 2014-04-03

**Authors:** Huitao Zhao, Mengxia Lu, Junmei Zuo

**Affiliations:** Department of Mathematics and Information Science, Zhoukou Normal University, Zhoukou, Henan 466001, China

## Abstract

A controlled model for a financial system through washout-filter-aided dynamical feedback control laws is developed, the problem of anticontrol of Hopf bifurcation from the steady state is studied, and the existence, stability, and direction of bifurcated periodic solutions are discussed in detail. The obtained results show that the delay on price index has great influences on the financial system, which can be applied to suppress or avoid the chaos phenomenon appearing in the financial system.

## 1. Introduction

For the last two decades, there have been growing interests in studying the complex dynamics of financial systems in both micro- and macroeconomics [1, 2]. It is well known that the economic activity is a complex human behavior; it has many uncertainties, which is reflected in the nonlinear model for economic dynamics such as Goodwin's nonlinear accelerator model [3], forced van der Pol model on business cycle [4], the dynamic IS-LM model [5], and nonlinear dynamical model on finance system [6–9]. In these models, chaotic phenomena are common. However, in economic activities, chaos is undesired sometimes, so we want to control the chaotic orbits to a stable state or a periodic orbit. For example, in [9, 10], the authors showed that the chaotic behavior of a microeconomic model can be stabilized to various periodic orbits by means of time-delayed feedback control.

On the other hand, delays are ubiquitous in life, so it is in the social and economic activities. There are at least two ways that time delays emerge in the dynamics of economic variables. One is the time lag between the time economic decisions are made and the time the decisions bear fruit [11]. The other is the behavior of economic agents known as rational expectations [12]. So, it is very meaningful to investigate the effects of time delay on economic activity [10].

The aim of this paper is to investigate the dynamics of a financial system by considering the effect of washout filters with time delay. By analyzing the characteristic equation of linearization of the system, we theoretically prove that the Hopf bifurcations occur in the model with delay. Furthermore, by using the theory of functional differential equation and Hassard's method [13], we also give the conditions to determine the direction and stability of the bifurcating periodic solutions. Finally, numerical results are given to support the theoretical prediction.

The rest of the paper is organized as follows. In Section 2, we propose the controlled finance system through washout filters with delay. In Section 3, we study the local stability and Hopf bifurcation of the equilibria. In Section 4, using the normal form theory and the center manifold reduction, explicit formulae are derived to determine the direction of bifurcation and the stability and other properties of bifurcating periodic solutions. In Section 5, we will give some numerical simulations to support the theoretical prediction. In Section 6, a brief discussion is given.

## 2. The Model

In [6, 7], the authors have reported a dynamical model of financial system composed of four subblocks: production, money, stock, and labor force. By setting proper dimensions and choosing appropriate coordinates, the authors have offered the simplified financial model which describes the time variation of three variables: the interest rate *x*, the investment demand *y*, and the price index *z*. The model is represented by three-dimensional ODEs:

(1) x˙=z+x(y−a),y˙=1−by−x2,z˙=−x−cz,

where *a* > 0 is the saving amount, *b* > 0 is the cost per investment, and *c* > 0 is the elasticity of demand of commercial market. This model is well studied in [6–9]; their results show that system (1) has abundant dynamical behaviors including Hopf bifurcation and chaos; however, the effect of time delay on the dynamics of this financial system was not taken into account.

In the following, we consider the effect of washout filters with time delay. We first consider a general form of dynamical system:

(2) $$\begin{array}{c} \dot{X}=f\left(X;\mu \right), \end{array}$$

where *X* is a vector and *μ* is a parameter. The washout-filter-aided controller assumes the following structure:

(3) X˙=f(X;μ)+u,w˙=Xi−dw,u=g(ρ;K),

where *u* is a control input, *g* is a control function, and *d* is the washout filter time constant. The following constraints should be fulfilled: *d* > 0, which guarantees the stability of the washout filter; *g*(0; *K*) = 0, which preserves the original equilibrium points.

In this paper, the controlled system is designed as follows:

(4) x˙(t)=z(t)+x(t)(y(t)−a),y˙(t)=1−by(t)−x2(t),z˙(t)=−x(t)−cz(t)+u(t),u˙(t)=k(z(t)−z(t−τ))−du(t),

where *k* > 0 is a control gain, *d* > 0 is an accommodation coefficient, and *τ* > 0 is time delay. *u* is the control input; differing from the time-delayed feedback controller (DFC) [10], the changing rate of controller *u* is influenced by the time delay feedback on price index *z* and adjusted by *u*. This system has the similar character with washout filter controller, *d* > 0, which guarantees the stability of the controller, and the original equilibrium points were preserved [14, 15].

## 3. Existence of Hopf Bifurcation

In this section, we choose the gain *k* as a constant and investigate the effect of time delay *τ* on the dynamic behavior of the controlled system (4). First, the following conclusions for the uncontrolled system (1) are needed.

**Lemma**

When *c* − *b* − *abc* ⩽ 0, that is, 1 + *ac* − (*c*/*b*)⩾0, system (1) has a unique equilibrium *P*_0_(0, 1/*b*, 0).

**Lemma**

When *c* − *b* − *abc* > 0, that is, 1 + *ac* − (*c*/*b*) < 0, system (1) has three equilibria *P*_0_(0, 1/*b*, 0) and $P_{\pm }(\pm \sqrt{\left(c-b-abc\right)/c},\left(1+ac\right)/c,\mp \left(1/c\right)\sqrt{\left(c-b-abc\right)/c})$.

The characteristic equation of the Jacobian matrix at the equilibria *P*_±_ of system (1) is

(5) $$\begin{array}{c} \lambda^{3}+a_{1}\lambda^{2}-a_{2}\lambda +a_{3}=0, \end{array}$$

where *a*_1_ = (*c* ^2^ + *bc* − 1)/*c*, *a*_2_ = (−*bc* ^2^ − 2*c* + 3*b* + 2*abc*)/*c*, and *a*_3_ = −2*b* + 2*c* − 2*abc*. Then, from Routh-Hurwitz criterion, the real parts of all the roots of the above equation are negative if and only if the conditions

(H1) *a*_1_ > 0, *a*_3_ > 0, and *a*_1_ *a*_2_ − *a*_3_ > 0 hold.

**Lemma 3**

- The equilibrium *P*_0_(0, 1/*b*, 0) of system (1) is stable when 1 + *ac* − (*c*/*b*) > 0 and *c* + *a* − (1/*b*) > 0.
- The equilibria *P*_±_ of system (1) are stable when *c* − *b* − *abc* > 0 and (H1) hold.

### 3.1. Hopf Bifurcation from the Stable Equilibrium *P*_0_

The linear equation of the controlled system (4) at *P*_0_ (where *u*_0_ = 0) is

(6) x˙(t)=z(t)−(a−1b)x(t),y˙(t)=−by(t),z˙(t)=−x(t)−cz(t)+u(t),u˙(t)=k(z(t)−z(t−τ))−du(t).

The associated characteristic equation of the linearized system is

(7) $$\begin{array}{c} \det\left(\begin{array}{cccc} \lambda +a-\frac{1}{b} & 0 & -1 & 0\\ 0 & \lambda +b & 0 & 0\\ 1 & 0 & \lambda +c & -1\\ 0 & 0 & k\left(e^{-\lambda \tau }-1\right) & \lambda +d \end{array}\right)=0. \end{array}$$

That is,

(8) $$\begin{array}{c} \left(\lambda +b\right)\left[\lambda^{3}+p_{1}\lambda^{2}+p_{2}\lambda +p_{3}+\left(q_{1}\lambda +q_{2}\right)e^{-\lambda \tau }\right]=0, \end{array}$$

where

(9) p1=a+c+d−1b,p2=(a−1b)(c+d)+cd−k+1,p3=(a−1b)(cd−k)+d,  q1=k,  q2=k(a−1b).

It is well known that the equilibrium *P*_0_(0, 1/*b*, 0,0) is stable if all the roots of (8) have negative real parts. Obviously, (8) always has a negative root *λ* = −*b*, for all *τ*⩾0, so, we only need to investigate the third transcendental polynomial equation:

(10) $$\begin{array}{c} \lambda^{3}+p_{1}\lambda^{2}+p_{2}\lambda +p_{3}+\left(q_{1}\lambda +q_{2}\right)e^{-\lambda \tau }=0. \end{array}$$

Obviously, if *λ* = ±*iω*  (*ω* > 0) is a pair of pure imaginary roots of (8), then *ω* satisfies

(11) −iω3−p1ω2+ip2ω+p3  +(iq1ω+q2)(cos⁡ωτ−isinωτ)=0.

Separating the real and imaginary parts, we have

(12) $$\begin{array}{c} \omega^{3}-p_{2}\omega =q_{1}\omega \cos\omega \tau -q_{2}\sin\omega \tau ,\\ p_{1}\omega^{2}-p_{3}=q_{1}\omega \sin\omega \tau +q_{2}\cos\omega \tau , \end{array}$$

and it follows that

(13) $$\begin{array}{c} \omega^{6}+p\omega^{4}+q\omega^{2}+r=0, \end{array}$$

where

(14) p=p12−2p2,  q=p22−2p1p3−q12,r=p32−q22.

Let *ζ* = *ω* ^2^, and (13) becomes

(15) $$\begin{array}{c} \zeta^{3}+p\zeta^{2}+q\zeta +r=0. \end{array}$$

Denote *h*(*ζ*) = *ζ* ^3^ + *pζ* ^2^ + *qζ* + *r*. We have the following.

**Lemma 4**

For (15), one has the following results:

- if *r* < 0, then (15) has at least one positive root;
- if *r*⩾0 and Δ = *p* ^2^ − 3*q* ⩽ 0, then (15) has no positive root;
- if *r*⩾0 and Δ = *p* ^2^ − 3*q* > 0, then (15) has positive root if and only if  $\zeta_{1}=\left(1/3\right)(-p+\sqrt{\Delta })>0$ and *h*(*ζ*_1_) ⩽ 0.

Without loss of generality, suppose that *ζ*_*i*_  (*i* = 1,2, 3) are positive roots of (15). Then, $\omega_{i}=\sqrt{\zeta_{i}}$ is a root of (13). From (12), we have

(16) τij=1ωi{arccosq1ω4+(p1q2−p2q1)ω2−q2p3q12ω2+q22+2jπ},                   j=0,1,2,….

Denote

(17) $$\begin{array}{c} \tau_{0}=\min\left\{\tau_{i}^{0}\;|\;i=1,2,3\right\}. \end{array}$$

Substituting *λ*(*τ*) into (10) and taking the derivative with respect to *τ*, we have

(18) $$\begin{array}{c} {\left[\frac{d\lambda }{d\tau }\right]}^{-1}=\frac{\left(3\lambda^{2}+2p_{1}\lambda +p_{2}\right)e^{\lambda \tau }+q_{1}}{\lambda \left(q_{1}\lambda +q_{2}\right)}-\frac{\tau }{\lambda }. \end{array}$$

From (12) and (18), through tedious computing, we get

(19) $$\begin{array}{c} {\left[\frac{d\lambda }{d\tau }\right]}_{\tau =\tau_{i}^{j}}^{-1}=\frac{\zeta_{i}}{\Lambda }h^{\prime }\left(\zeta_{i}\right), \end{array}$$

where Λ = *ω*_*i*_^2^(*q*_1_ *ω*_*i*_^2^ + *q*_2_). Since *ζ*_*i*_ > 0, then [*dλ*/*dτ*]_*τ*=*τ*_*i*_^*j*^_^−1^ and *h*′(*ζ*_*i*_) have the same sign. Thus, from Lemmas 3 and 4, we have the following theorem.

**Theorem 5**

Suppose 1 + *ac* − (*c*/*b*) > 0 and *c* + *a* − (1/*b*) > 0; then, one has the following:

- if *r*⩾0 and Δ = *p* ^2^ − 3*q* ⩽ 0, then the equilibrium *P*_0_ is stable for all *τ*⩾0;
- if *r* < 0 (or *r*⩾0 and Δ = *p* ^2^ − 3*q* > 0) and *h*′(*ζ*_*i*_) ≠ 0, then, when *τ* ∈ [0, *τ*_0_), the equilibrium *P*_0_ is stable, and system (4) undergoes Hopf bifurcation at *P*_0_ when *τ* passes through *τ*_0_.

### 3.2. Hopf Bifurcation from the Stable Equilibria *P*_±_

In this subsection, we assume that system (1) has two stable equilibria $P_{\pm }(\pm \sqrt{\left(c-b-abc\right)/c},\left(1+ac\right)/c,\mp \left(1/c\right)\sqrt{\left(c-b-abc\right)/c})$. Due to the symmetry of *P*_+_ and *P*_−_, it is sufficient to analyze the stability of *P*_+_.

Let $P_{+}=(\tilde{x},\tilde{y},\tilde{y})=\left(\sqrt{\left(c-b-abc\right)/c},\left(1+ac\right)/c,-\left(1/c\right)\sqrt{\left(c-b-abc\right)/c}\right)$. By the linear transforms $(t)=x(t)-\tilde{x}$, $Y(t)=y(t)-\tilde{y}$, $Z(t)=z(t)-\tilde{z}$, and *U*(*t*) = *u*(*t*), the linear equation of the controlled system (4) at *P*_+_ is

(20) X˙(t)=Z(t)−(a−y~)X(t)+x~Y(t),Y˙(t)=−bY(t)−2x~X(t),Z˙(t)=−X(t)−cZ(t)+U(t),U˙(t)=k(Z(t)−Z(t−τ))−dU(t).

The associated characteristic equation of system (20) is

(21) $$\begin{array}{c} \det\left(\begin{array}{cccc} \lambda +a-\tilde{y} & -\tilde{x} & -1 & 0\\ 2\tilde{x} & \lambda +b & 0 & 0\\ 1 & 0 & \lambda +c & -1\\ 0 & 0 & k\left(e^{-\lambda \tau }-1\right) & \lambda +d \end{array}\right)=0. \end{array}$$

Expand (21), and we have

(22) $$\begin{array}{c} \lambda^{4}+\rho_{1}\lambda^{3}+\rho_{2}\lambda^{2}+\rho_{3}\lambda +\rho_{4}+\left(\sigma_{1}\lambda^{2}+\sigma_{2}\lambda +\sigma_{3}\right)e^{-\lambda \tau }=0, \end{array}$$

where

(23) ρ1=a+b+c+d−y~,ρ2=b(a−y~)+2x~2+cd−k+(c+b−y~)(c+d),ρ3=(a+c−y~)(cd−k)+(c+d)(ab−by~+2x~2),ρ4=(ab−by~+2x~2)(cd−k),σ1=k,  σ2=k(a+b−y~),σ3=k(ab−by~+2x~2).

Suppose *iω* is a root of (22); then, *ω* satisfies

(24) ω4−ρ2ω2+ρ4=(σ1ω2−σ3)cos⁡ωτ−σ2ωsinωτ,ρ1ω3−ρ3ω=σ2ωcos⁡ωτ+(σ1ω2−σ3)sinωτ,

which lead to

(25) $$\begin{array}{c} \omega^{8}+\kappa_{1}\omega^{6}+\kappa_{2}\omega^{4}+\kappa_{3}\omega^{2}+\kappa_{4}=0, \end{array}$$

where

(26) $$\begin{array}{c} \kappa_{1}=\rho_{1}^{2}-2\rho_{2},\;\;\kappa_{2}=\rho_{2}^{2}+2\rho_{4}-2\rho_{1}\rho_{3}-\sigma_{1}^{2},\\ \kappa_{3}=\rho_{3}^{2}-2\rho_{2}\rho_{4}+2\sigma_{1}\sigma_{3}-\sigma_{2}^{2},\;\;\kappa_{4}=\rho_{4}^{2}-\sigma_{3}^{2}. \end{array}$$

Denote *z* = *ω* ^2^; then, (24) becomes

(27) $$\begin{array}{c} z^{4}+\kappa_{1}z^{3}+\kappa_{2}z^{2}+\kappa_{3}z+\kappa_{4}=0. \end{array}$$

Denote

(28) $$\begin{array}{c} g\left(z\right)=z^{4}+\kappa_{1}z^{3}+\kappa_{2}z^{2}+\kappa_{3}z+\kappa_{4}. \end{array}$$

Clearly, if *κ*_4_ < 0, then (27) has at least one positive root. Suppose *z*_*k*_ is a positive root of (27); then, $\omega_{k}=\sqrt{z_{k}}$ is a root of (25). From (24), we have

(29) τk(j)=1ωk{arccos((σ1ωk6−(ρ2σ1−ρ1σ2+σ3)ωk4        +(ρ4σ1+ρ2σ3−ρ3σ2)ωk2−ρ4σ3)       ×((σ1ωk2−σ3)2+σ22ωk2)−1)  +2jπ}, j=0,1,2,….

Substituting *λ*(*τ*) into (22) and taking the derivative with respect to *τ*, we obtain

(30) [dλdτ]−1 =(4λ3+3ρ1λ2+2ρ2λ+ρ3)eλτ+2σ1λ+σ2λ(σ1λ2+σ2λ+σ3)  −τλ.

From (24) and (30), we have

(31) $$\begin{array}{c} {\left[\frac{d\lambda }{d\tau }\right]}_{\tau =\tau_{k}^{j}}^{-1}=\frac{z_{k}}{\Gamma }g^{\prime }\left(z_{k}\right), \end{array}$$

where Γ = *ω*_*k*_^2^((*σ*_3_ − *σ*_1_ *ω*_*k*_^2^)^2^ + *σ*_2_^2^ *ω*_*k*_^2^). Thus, from the above analysis, we have the following.

**Theorem 6**

Suppose *κ*_4_ < 0 and *g*′(*z*_*k*_) ≠ 0; then, system (4) undergoes Hopf bifurcation at the steady state *P*_±_ when *τ* passes through *τ*_*k*_^(*j*)^.

## 4. Direction and Stability of the Hopf Bifurcation

In Section 3, we obtain the conditions under which a family of periodic solutions bifurcate from the steady state at the critical value of *τ*. In this section, following the ideal of [13], we derive the explicit formulae for determining the properties of the Hopf bifurcation at the critical value of *τ* using the normal form and the center manifold theory.

In this section, we always assume that system (4) undergoes Hopf bifurcation at the steady state (x*, y*, z*) for *τ* = *τ*_*i*_, and then ±*iω*_*i*_ is the corresponding purely imaginary roots of the characteristic equation at the steady state (x*, y*, z*).

Let *u*_1_ = *x* − x*, *u*_2_ = *y* − y*, *u*_3_ = *z* − z*, *u*_4_ = *u*, $\bar{u_{i}}=u_{i}(\tau t)$, and *τ* = *τ*_*i*_ + *μ* and drop the bars for simplification of notations. Then, system (4) can be rewritten as a functional differential equation in *ℂ*([−1,0], ℝ^4^):

(32) $$\begin{array}{c} \dot{u}\left(t\right)=L_{\mu }\left(u_{t}\right)+f\left(\mu ,u_{t}\right), \end{array}$$

where *u* = (*u*_1_, *u*_2_, *u*_3_, *u*_4_)^*T*^. For *ϕ* = (*ϕ*_1_, *ϕ*_2_, *ϕ*_3_, *ϕ*_4_)^*T*^ ∈ *ℂ*([−1,0], ℝ^4^),

(33) Lμ(ϕ)=(τi+μ)[−a+y∗x∗102x∗−b00−10−c100k−d][ϕ1(0)ϕ2(0)ϕ3(0)ϕ4(0)]+(τi+μ)[00000000000000−k0][ϕ1(−1)ϕ2(−1)ϕ3(−1)ϕ4(−1)],f(μ,ϕ)=(τi+μ)[ϕ1(0)ϕ2(0)ϕ12(0)00].

Obviously, *L*(*μ*) is a continuous linear function mapping *ℂ*([−1,0], ℝ^4^) into ℝ^4^. By the Riesz representation theorem, there exists a 4 × 4 matrix function *η*(*θ*, *μ*)  (−1 ⩽ *θ* ⩽ 0), whose elements are of bounded variation such that

(34) $$\begin{array}{c} L_{\mu }\phi =\overset{0}{\underset{-1}{\int }}d\eta \left(\theta ,0\right)\phi \left(\theta \right),\;\text{for}\;\phi \in \mathbb{C}\left(\left[-1,0\right],{\mathbb{R}}^{4}\right). \end{array}$$

In fact, we can choose

(35) η(θ,μ)=(τi+μ)[−a+y∗x∗102x∗−b00−10−c100k−d]δ(θ)+(τi+μ)[00000000000000−k0]δ(θ+1),

where *δ* denote Dirac-delta function. For *ϕ* ∈ *ℂ*([−1,0], ℝ^4^), define

(36) A(μ)ϕ(θ)={dϕ(θ)dθ,θ∈[−1,0),∫−10dη(s,μ)ϕ(s),θ=0,R(μ)ϕ(θ)={0,θ∈[−1,0),f(μ,ϕ),θ=0.

Then, when *θ* = 0, the system

(37) $$\begin{array}{c} {\dot{u}}_{t}=A\left(\mu \right)u_{t}+R\left(\mu \right)u_{t} \end{array}$$

is equivalent to the system (32), where *u*_*t*_(*θ*) = *u*(*t* + *θ*) and *θ* ∈ [−1,0]. For *ψ* ∈ *ℂ* ^1^([0,1], (ℝ^4^)*), define

(38) $$\begin{array}{c} A^{*}\psi \left(s\right)=\{\begin{array}{cc} -\frac{d\psi \left(s\right)}{ds}, & s\in \left(0,1\right],\;\\ \overset{0}{\underset{-1}{\int }}d\eta^{T}\left(t,0\right)\psi \left(-t\right), & s=0, \end{array} \end{array}$$

and a bilinear inner product

(39) 〈ψ(s),ϕ(θ)〉=ψ¯(0)ϕ(0)−∫−10∫ξ=0θψ¯(ξ−θ)dη(θ)ϕ(ξ)dξ,

where *η*(*θ*) = *η*(*θ*, 0); let *A* = *A*(0); then, *A* and A* are adjoint operators. By the discussion in Section 3, we know that ±*iω*_*i*_ *τ*_*i*_ are eigenvalues of *A*. Thus, they are also eigenvalues of A*. We first need to compute the eigenvector of *A* and A* corresponding to *iω*_*i*_ *τ*_*i*_ and −*iω*_*i*_ *τ*_*i*_, respectively.

Suppose that *q*(*θ*) = (1, *α*, *β*, *γ*)^*T*^ *e* ^*iω*_*i*_*τ*_*i*_*θ*^ is the eigenvector of *A* corresponding to *iω*_*i*_ *τ*_*i*_. Then, *Aq*(*θ*) = *iω*_*i*_ *τ*_*i*_ *q*(*θ*). It follows from the definition of *A*, *L*_*μ*_ *ϕ*, and *η*(*θ*, *μ*) that

(40) τi[iωi+a−y∗−x∗−10−2x∗iωi+b0010iωi+c−100−k+ke−iωiτiiωi+d]q(0)  =[0000].

Thus, we can easily obtain *α* = 2x*/(*iω*_*i*_ + *b*), *β* = *iω*_*i*_ + *a* − y* − αx*, *γ* = 1 + (*iω*_*i*_ + *c*)*β*, and *q*(0) = (1, *α*, *β*, *γ*)^*T*^.

Similarly, let q*(*s*) = *D*(1, α*, β*, γ*)*e* ^*iω*_*i*_*τ*_*i*_*s*^ be the eigenvector of A* corresponding to −*iω*_*i*_ *τ*_*i*_. By the definition of A*, we can compute α* = −x*/(*iω*_*i*_ − *b*), β* = *iω*_*i*_ − *a* + y* + 2x*α*, and γ* = −(β*/(*iω*_*i*_ − *d*)).

In order to assure that 〈q*(*s*), *q*(*θ*)〉 = 1, we need to determine the value of *D*. From (39), we have

(41) 〈q∗(s),q(θ)〉=D¯(1,α∗¯,β∗¯,γ∗¯)(1,α,β,γ)T −∫−10∫ξ=0θD¯(1,α∗¯,β∗¯,γ∗¯)e−iωiτi(ξ−θ)dη(θ)      ×(1,α,β,γ)Teiωiτiξdξ=D¯{1+αα∗¯+ββ∗¯+γγ∗¯     −∫−10(1,α∗¯,β∗¯,γ∗¯)θeiθωiτidη(θ)(1,α,β,γ)T}=D¯{1+αα∗¯+ββ∗¯+γγ∗¯−kβγ∗¯e−iωiτi}.

Thus, we can choose

(42) $$\begin{array}{c} \bar{D}={\left\{1+\alpha \bar{\alpha^{*}}+\beta \bar{\beta^{*}}+\gamma \bar{\gamma^{*}}-k\beta \bar{\gamma^{*}}e^{-i\omega_{i}\tau_{i}}\right\}}^{-1}, \end{array}$$

such that 〈q*(*s*), *q*(*θ*)〉 = 1, $\langle q^{*}(s),\bar{q}(\theta )\rangle =0$.

In the following, we first compute the coordinates to describe the center manifold *C*_0_ at *μ* = 0. Define

(43)     z(t)=〈q∗,ut〉,  W(t,θ)=ut(θ)−2Re{z(t)q(θ)}.

On the center manifold *C*_0_, we have

(44) W(t,θ)=W(z(t),z¯(t),θ)=W20(θ)z22+W11(θ)zz¯+W02(θ)z¯22 +W30(θ)z36+⋯,

where *z* and $\bar{z}$ are local coordinates for center manifold *C*_0_ in the direction of *q* and $\bar{q}$. Note that *W* is real if *u*_*t*_ is real. We consider only real solutions. For the solution *u*_*t*_ ∈ *C*_0_, since *μ* = 0, we have

(45) z˙=iωiτiz +〈q∗(θ),f(0,W(z(t),z¯(t),θ)+2Re{z(t)q(θ)})〉=iωiτiz +q∗¯(0)f(0,W(z(t),z¯(t),0)+2Re{z(t)q(0)})≜iωiτiz+q∗¯(0)f0(z,z¯)=iωiτiz+g(z,z¯),

where

(46) g(z,z¯)=q∗¯(0)f0(z,z¯)=g20(θ)z22+g11(θ)zz¯+g02(θ)z¯22 +g21(θ)z2z¯2+⋯.

From (43) and (44), we have

(47) ut(θ)=(u1t(θ),u2t(θ),u3t(θ),u4t(θ))T=W(t,θ)+zq(θ)+z¯ q¯(θ).

In addition, *q*(*θ*) = (1, *α*, *β*, *γ*)^*T*^ *e* ^*iω*_*i*_*τ*_*i*_*θ*^; then,

(48) u1t(0)=z+z¯+W20(1)(0)z22+W11(1)(0)zz¯+W02(1)(0)z¯22+O(|(z,z¯)|3),u2t(0)=αz+α¯ z¯+W20(2)(0)z22+W11(2)(0)zz¯+W02(2)(0)z¯22+O(|(z,z¯)|3),u3t(0)=βz+β¯z¯+W20(3)(0)z22+W11(3)(0)zz¯+W02(3)(0)z¯22+O(|(z,z¯)|3),u4t(0)=γz+γ¯ z¯+W20(4)(0)z22+W11(4)(0)zz¯+W02(4)(0)z¯22+O(|(z,z¯)|3).

By the definition of *f*(*μ*, *x*_*t*_), we have

(49) $$\begin{array}{c} g\left(z,\bar{z}\right)=\bar{D}\tau_{i}\left(1,\bar{\alpha^{*}},\bar{\beta^{*}},\bar{\gamma^{*}}\right)\left[\begin{array}{c} u_{1t}\left(0\right)u_{2t}\left(0\right)\\ u_{1t}^{2}\left(0\right)\\ 0\\ 0 \end{array}\right]. \end{array}$$

Substituting *u*_1*t*_(0), *u*_2*t*_(0), *u*_3*t*_(0), and *u*_4*t*_(0) into the above equation and comparing the coefficients with (46), we get

(50) g20=2D¯τi(α+α∗¯),g11=D¯τi(α+α¯+2α∗¯),g02=2D¯τi(α¯+α∗¯),g21=D¯τi[2W11(2)(0)+W20(2)(0)+2αW11(1)(0)   +α¯W20(1)(0)+2α∗¯(2W11(1)(0)+W20(1)(0))].

In order to assure the value of *g*_21_, we need to compute *W*_20_(*θ*) and *W*_11_(*θ*). From (37) and (43), we have

(51) W˙=u˙t−z˙q−z¯˙ q¯={AW−2Re{q¯∗(0)f0q(θ)},θ∈[0,1)AW−2Re{q¯∗(0)f0q(θ)}+f0,θ=0≜AW+H(z,z¯,θ),

where

(52) $$\begin{array}{c} H\left(z,\bar{z},\theta \right)=H_{20}\left(\theta \right)\frac{z^{2}}{2}+H_{11}\left(\theta \right)z\bar{z}+H_{02}\left(\theta \right)\frac{{\bar{z}}^{2}}{2}+\cdots . \end{array}$$

Notice that, near the origin on the center manifold *C*_0_, we have

(53) $$\begin{array}{c} \dot{W}=W_{z}\dot{z}+W_{\bar{z}}\dot{\bar{z}}; \end{array}$$

thus, we have

(54) $$\begin{array}{c} \left(A-2i\omega_{k}\tau_{k}I\right)W_{20}\left(\theta \right)=-H_{20}\left(\theta \right),\\ \;\;AW_{11}\left(\theta \right)=-H_{11}\left(\theta \right). \end{array}$$

Since (51), for *θ* ∈ [−1,0), we have

(55) H(z,z¯,θ)=−q∗¯(0)f0q(θ)−q∗(0)f¯0q¯(θ)=−gq(θ)−g¯ q¯(θ).

Comparing the coefficients with (51) gives that

(56) $$\begin{array}{c} H_{20}\left(\theta \right)=-g_{20}q\left(\theta \right)-{\bar{g}}_{02}\bar{q}\left(\theta \right),\\ \;H_{11}\left(\theta \right)=-g_{11}q\left(\theta \right)-{\bar{g}}_{11}\bar{q}\left(\theta \right). \end{array}$$

From (54), (56), and the definition of *A*, we can get

(57) $$\begin{array}{c} {\dot{W}}_{20}\left(\theta \right)=2i\omega_{i}\tau_{i}W_{20}\left(\theta \right)+g_{20}q\left(\theta \right)+{\bar{g}}_{02}\bar{q}\left(\theta \right). \end{array}$$

Notice that *q*(*θ*) = *q*(0)*e* ^*iω*_*i*_*τ*_*i*_*θ*^, and we have

(58) $$\begin{array}{c} W_{20}\left(\theta \right)=\frac{ig_{20}}{\omega_{i}\tau_{i}}q\left(0\right)e^{i\omega_{i}\tau_{i}\theta }+\frac{i{\bar{g}}_{02}}{3\omega_{i}\tau_{i}}\bar{q}\left(0\right)e^{-i\omega_{i}\tau_{i}\theta }+E_{1}e^{2i\omega_{i}\tau_{i}\theta }, \end{array}$$

where *E*_1_ = (*E*_1_^(1)^, *E*_1_^(2)^, *E*_1_^(3)^, *E*_1_^(4)^)^*T*^ ∈ ℝ^4^ is a constant vector. In the same way, we can also obtain

(59) $$\begin{array}{c} W_{11}\left(\theta \right)=-\frac{ig_{11}}{\omega_{i}\tau_{i}}q\left(0\right)e^{i\omega_{i}\tau_{i}\theta }+\frac{i{\bar{g}}_{11}}{\omega_{i}\tau_{i}}\bar{q}\left(0\right)e^{-i\omega_{i}\tau_{i}\theta }+E_{2}, \end{array}$$

where *E*_2_ = (*E*_2_^(1)^, *E*_2_^(2)^, *E*_2_^(3)^, *E*_2_^(4)^)^*T*^ ∈ ℝ^4^ is also a constant vector.

In what follows, we will compute *E*_1_ and *E*_2_. From the definition of *A* and (54), we have

(60) ∫−10dη(θ)W20(θ)=2iωiτiW20(0)−H20(0),

(61) ∫−10dη(θ)W11(θ)=−H11(0).

From (51) and (52), we have

(62) $$\begin{array}{c} H_{20}\left(0\right)=-g_{20}q\left(0\right)-{\bar{g}}_{02}\bar{q}\left(0\right)+2\tau_{i}{\left[\alpha ,1,0,0\right]}^{T}, \end{array}$$

(63) H11(0)=−g11q(0)−g¯11q¯(0)+2τi[Re{α},1,0,0]T.

Substituting (58) and (62) into (60) and noticing that

(64) $$\begin{array}{c} \left[i\omega_{i}\tau_{i}I-\overset{0}{\underset{-1}{\int }}e^{i\omega_{i}\tau_{i}\theta }d\eta \left(\theta \right)\right]q\left(0\right)=0,\\ \left[-i\omega_{i}\tau_{i}I-\overset{0}{\underset{-1}{\int }}e^{-i\omega_{i}\tau_{i}\theta }d\eta \left(\theta \right)\right]\bar{q}\left(0\right)=0, \end{array}$$

we obtain

(65) [2iωi+a−y∗−x∗−10−2x∗2iωi+b00102iωi+c−100−k+ke−iωiτi2iωi+d]E1  =2τi[α100].

That is,

(66) E1=2τi[2iωi+a−y∗−x∗−10−2x∗2iωi+b00102iωi+c−100−k+ke−iωiτi2iωi+d]−1×[α100].

Similarly, substituting (59) and (63) into (61), we can get the formula of *E*_2_, where

(67) E2=2τi[a−y∗−x∗−10−2x∗b0010c−100−kd]−1[Re{α}100].

Thus, we can determine *W*_20_(*θ*) and *W*_11_(*θ*). Furthermore, we can determine each *g*_*ij*_. Therefore, each *g*_*ij*_ is determined by the parameters and delay in (4). Thus, we can compute the following values:

(68) c1(0)=i2ωiτi(g20g11−2|g11|2−13|g02|2)+12g21,μ2=−Re{c1(0)}Re{λ′(0)},T2=−Im⁡{c1(0)}+μ2Im⁡{λ′(0)}ωiτi,β2=2Re{c1(0)},

which determine the quantities of bifurcating periodic solutions in the center manifold at the critical value *τ*_*i*_; that is, *μ*_2_ determines the directions of the Hopf bifurcation; if *μ*_2_ > 0 (<0), then the Hopf bifurcation is supercritical (subcritical) and the bifurcation exists for *τ* > *τ*_*i*_ (<*τ*_*i*_); *β*_2_ determines the stability of the bifurcation periodic solutions; the bifurcating periodic solutions are stable (unstable) if *β*_2_ < 0 (>0); and *T*_2_ determines the period of the bifurcating periodic solutions: the period increases (decreases) if *T*_2_ > 0 (<0).

## 5. Numerical Simulation

In this section, we present some numerical results to verify the analytical predictions obtained in the previous section. These numerical simulation results constitute excellent validations of our theoretical analysis; it is shown that the chaotic orbit can be controlled to a periodic orbit by using washout-filter-aided controller with time delay.

**Figure 1:**
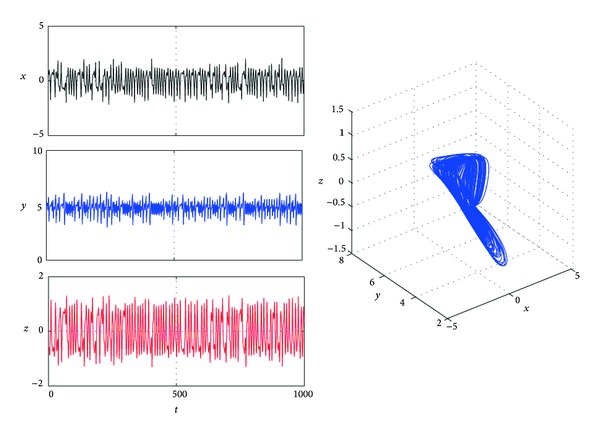
Trajectories *x*(*t*), *y*(*t*), and *z*(*t*) and phase graphs of system (1) with *a* = 4, *b* = 0.1, and *c* = 1.

Figure 1 shows that system (1) is chaotic when *a* = 4, *b* = 0.1, and *c* = 1.

### 5.1. Hopf Bifurcation from the Stable Equilibrium *P*_0_

**Figure 2:**
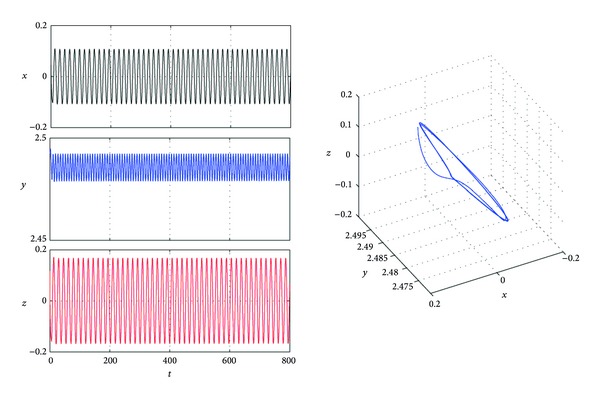
Trajectories *x*(*t*), *y*(*t*), and *z*(*t*) and phase graphs of system (4) with *a* = 4, *b* = 0.4, *c* = 1, *k* = 1, *d* = 1, and *τ* = 5.5940.

In this subsection, we choose *a* = 4, *b* = 0.4, and *c* = 1; then, system (1) has only a stable equilibrium *P*_0_(0,2.5,0). From the algorithm of Section 3, we get that *r* = (1.5*d* − 1.5*k*)^2^ − 2.25*k* ^2^; thus, if *k* > *d*/2, then *r* < 0. From Lemma 4, (15) has at least one positive root. Let *d* = 1 and *k* = 1. From the algorithm of Section 3, we can compute *τ*_0_≐5.5932. Thus, from Theorem 5, the equilibrium *P*_0_(0,2.5,0) is asymptotically stable when *τ* < *τ*_0_, and, as *τ* crosses *τ*_0_, there are periodic orbits bifurcating from *P*_0_(0,2.5,0) (Figure 2).

### 5.2. Hopf Bifurcation from the Stable Equilibria *P*_±_

**Figure 3:**
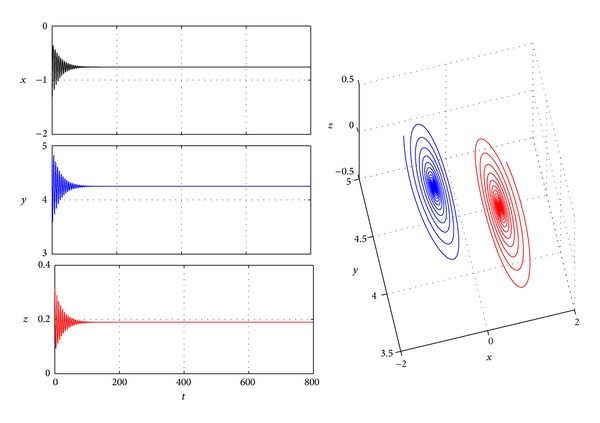
Trajectories *x*(*t*), *y*(*t*), and *z*(*t*) and phase graphs of system (1) with *a* = 4, *b* = 0.1, and *c* = 4.

**Figure 4:**
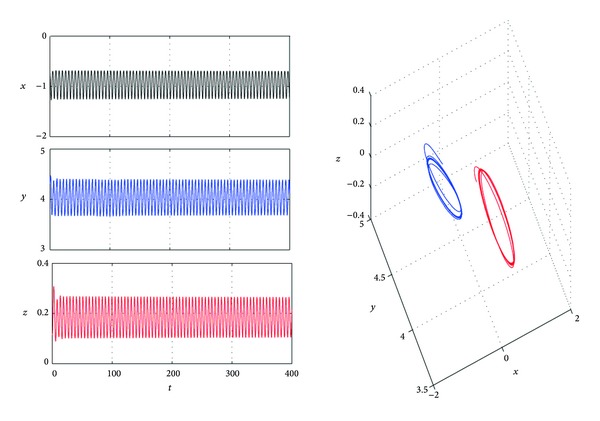
Trajectories *x*(*t*), *y*(*t*), and *z*(*t*) and phase graphs of system (4) with *a* = 4, *b* = 0.1, *c* = 4, *k* = 2, *d* = 1, and *τ* = 2.37.

In this subsection, we choose *a* = 4, *b* = 0.1, and *c* = 4; then, from Lemma 3, system (1) has three equilibria: *P*_0_(0,10,0) is unstable and *P*_±_(±0.7416,4.5, ∓0.3708) are stable (Figure 3). If we choose *k* = 2, and *d* = 1, from the algorithm of Section 3, we can get that the bifurcating value of *τ* is *τ*_*h*_ = 2.3542. When *τ* pass through *τ*_*h*_ = 2.3542, a family of periodic orbits will bifurcate from equilibria *P*_±_, respectively (Figure 4).

### 5.3. Application to Control of Chaos

**Figure 5:**
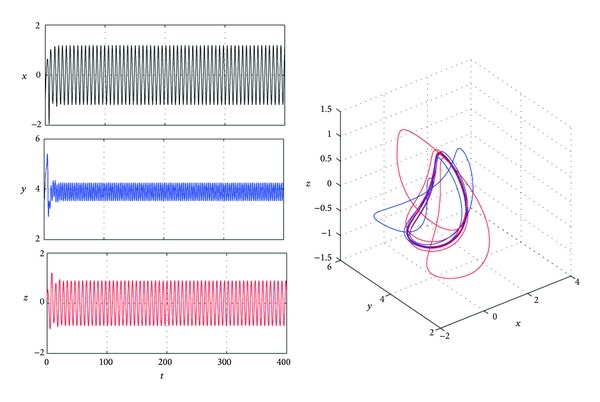
Trajectories *x*(*t*), *y*(*t*), and *z*(*t*) and phase graphs of system (4) with *a* = 4, *b* = 0.1, *c* = 1, *k* = 1, *d* = 1, and *τ* = 2.

From Figure 1, we can see that system (1) is chaotic when *a* = 4, *b* = 0.1, and *c* = 1. If we choose *k* = *d* = 1, a family of periodic orbits bifurcate from the equilibria of system (4) at some critical values of *τ*. This can be verified by Figure 5.

## 6. Conclusion

In this paper, we have investigated a financial system with time-delayed washout-filters-aided controller. Taking the time delay *τ* as bifurcating parameter, we discussed the conditions at which periodic orbits bifurcate from the equilibria *P*_0_ and *P*_±_, respectively. The stability and direction of bifurcated periodic solutions have been also investigated in detail. And the obtained results can be applied to control the chaos of this financial system.

From a financial sense, the obtained results show that the delay on price index has great influence on the financial system, which can be applied to suppress or avoid the chaos phenomenon appearing in the financial system, so as to make the economic system run well. On the other hand, the control gain *k* is also applied to influence the dynamical behaviors of this financial system; it will be investigated in the near future.
